# Current State of Carbohydrate Recognition and C-Type Lectin Receptors in *Pneumocystis* Innate Immunity

**DOI:** 10.3389/fimmu.2021.798214

**Published:** 2021-12-16

**Authors:** Theodore J. Kottom, Eva M. Carmona, Andrew H. Limper

**Affiliations:** ^1^ Thoracic Diseases Research Unit, Departments of Medicine and Biochemistry, Mayo Clinic, Rochester, MN, United States; ^2^ Department of Laboratory Medicine and Pathology, Mayo Clinic, Rochester, MN, United States

**Keywords:** beta-glucans, CLR, inflammation, *Pneumocystis*, pneumonia

## Abstract

*Pneumocystis jirovecii* is one of the most common fungal pathogens in immunocompromised individuals. Pneumocystis jirovecii pneumonia (PJP) causes a significant host immune response that is driven greatly by the organism’s cell wall components including β-glucans and major surface glycoprotein (Msg). These ligands interact with a number of C-type lectin receptors (CLRs) leading to downstream activation of proinflammatory signaling pathways. This minireview provides a brief overview summarizing known CLR/*Pneumocystis* interactions.

## Introduction

*Pneumocystis jirovecii* is the opportunistic fungal organism responsible for Pneumocystis jirovecii pneumonia (PJP) that causes severe morbidity and mortality in immunocompromised individuals and is one of the top 10 severe fungal infections in the world (1). *Pneumocystis* species are fungi that belong to the ascomycetes and have a diminutive trophic form and a larger cyst or asci form (2). Regarding the organism’s cell wall, both forms possess abundant major surface glycoproteins (Msgs) also termed glycoprotein A (gpA) (the genomes of *Pneumocystis* spp. encode for an abundance of Msgs proteins compromising approximately 3-6% of the total genome) (3). This large multicopy family is thought to be important for host/organism interactions as well as evasion of the host immune response (3). The cyst form also contains substantial amounts of β-glucans (4), and the fungus has the required enzymes for the synthesis and degradation of β-1,3- and β-1,6-linkages (4, 5). Furthermore, the *Pneumocystis* cell wall lacks chitin (3) a component of fungal organisms as well as α-glucans which are present in a number of pathogenic and nonpathogenic fungal organisms (6–8). Lastly, *Pneumocystis* lacks hyper mannose glycosylation on its outer surface unlike other pathogenic fungi such as *Candida albicans* (9) and its low complex composition hypothesized as a way to avoid host immune detection (3).

**Figure 1:**
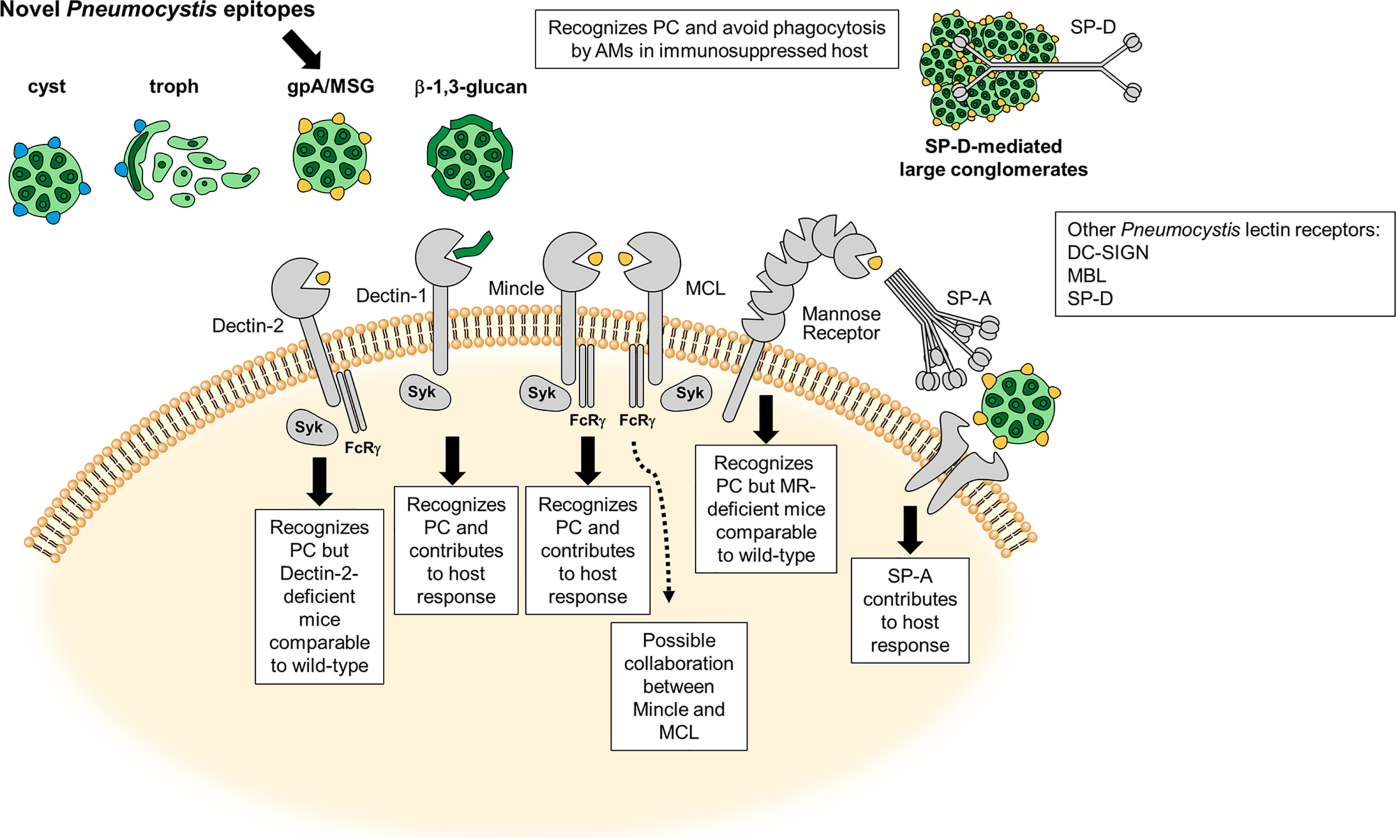
Carbohydrate Recognition and C-type Lectin (CLR) Receptors in *Pneumocystis* (PC). Schematic illustration adapted from Hoving (10) of the most characterized carbohydrate recognition receptors for the fungal organism and brief description of the host response. Major surface glycoprotein (Msg) is a novel ligand on the *Pneumocystis* cell surface. The dotted line represents a possible connection with MCL and Mincle on inflammatory signaling in the presence of *Pneumocystis* Msg (11).

The major *Pneumocystis* ligands β-glucans and Msgs, have been shown to interact with a number of known C-type lectin receptors (CLRs) and are discussed below (**Figure 1** and **Table 1**).

**Table 1:** List of host immune receptors, their *Pneumocystis* ligands, presence in relevant cell types in PCP, and importance in host/*Pneumocystis* interactions or response.

Receptor	Ligand	Cell Type	Importance	Reference
DC-SIGN (CD209)	Msg/gpA	DCs, alveolar macrophages (AMs)	++	(12–15)
Dectin-1 (CLEC7A)	B-1,3 glucan	macrophages, dendritic cells (DCs), bronchial epithelial cells, pulmonary epithelium	++	(16–21)
Dectin-2 (CLEC6A)	Msg/gpA	DCs, macrophages	+	(12, 22)
Mincle (CLEC4E)	Msg/gpA	Monocytes, macrophages, neutrophils, DCs	++	(12, 16)
MCL (CLEC4D)	Msg/gpA	Monocytes, macrophages, neutrophils, DCs	++ (*in vitro*)	(11, 12)
Mannose Binding Lectin (MBL) (COLEC1)	Msg/gpA	plasma	++	(23, 24)
Mannose Receptor (MR) (CD206)	Msg/gpA	AMs, DCs, monocytes	++ (in vitro)	(25–30)
Surfactant protein A (SP-A), Surfactant protein D (SP-D)	Msg/gpA	Lung lavage fluids	++	(31–33)
Surfactant protein B (SP-B)	(Binds organism?)	Lung lavage fluids	*	(34)

++, strong evidence for importance in host response/interactions to Pneumocystis; +, moderate evidence for importance host response to Pneumocystis; +, involved in Pneumocystis host response/interactions weak or further studies needed; *host response/interactions to Pneumocystis unknown.

## DC-Sign

Dendritic Cell-Specific Intercellular adhesion molecule-3-Grabbing Nonintegrin (DC-SIGN) is a CLR that has high affinity for fungal N-mannans (9, 35). Although highly recognized as an important CLR for many funga/host interactions, little is known about its role in *Pneumocystis* binding. Elsegeiny et al. showed that a human DC-SIGN Fc (fragment crystallizable) fusion could bind both cyst and trophic forms (15). This same lab shown that in immunodeficient humanized mice (huNOG-EXL) expressing high levels of DC-SIGN mRNA, there were significantly lower fungal numbers in the immunosuppressed state, suggesting the importance of the CLR in organism burden control (14). We and others have shown through Fc fusions of the DC-SIGN CLR that the Msg on the surface of *Pneumocystis* serves as a ligand for DC-SIGN (12, 13). To date, no studies have evaluated mutations of the 7 known distinct genes encoding the human DC-SIGN homolog using the Pneumocystis pneumonia (PCP) mouse model to further study the relevance of these mutations in contributing to *Pneumocystis* innate immunity (36).

## Dectin-1

CLR Dectin-1 has long been considered the preeminent fungal β-glucan receptor (37). Its role in *Pneumocystis* host defense was first demonstrated through its importance in nonopsonized phagocytosis of the fungal organism by the alveolar macrophage (AM) *in vitro* (21). The receptor appears to colocalize (bind to the β-glucan carbohydrate linkages) with the fungal cell wall and is important in killing of *Pneumocystis* organisms. Furthermore, competitive inhibition studies with *Saccharomyces cerevisiae* β-glucan rather than mannan demonstrated that organism killing by AMs could be significantly reduced, providing more credence to the importance of Dectin-1 in the control of organism clearance by AMs (21). Other have also shown that Dectin-1 colocalizes with TLR2 and mannose receptor (MR) in AMs (discussed below) challenged with *Pneumocystis* organisms (17). In addition to the importance of Dectin-1 in *Pneumocystis* and AM identification and killing, the receptor has also been shown to be important in dendritic cell (DC) interactions with *Pneumocystis*. Studies conducted by Carmona et al. show that human DCs preincubated with a monoclonal antibody to Dectin-1, can significantly reduce TNFa when stimulated with *Pneumocystis*-specific β-glucans (38).

A number of studies examined the role of Dectin-1 using *in vivo* models of PCP. First, Saijo et al. demonstrated that in the PCP rodent model, Dectin-1-deficient mice displayed significantly more cysts than wild type mice in both immunocompetent and immunosuppressed models of PCP, being the first to link Dectin-1 with importance in controlling organism burden in PCP (19). Secondly, an eloquent study by Rapaka et al., demonstrated that when severe combined immunodeficiency (SCID) mice with PCP were given an adenoviral expressing a Fc-Dectin-1 fusion, the construct reduced overall organism burden and lung parameters associated with organism/host response damage (20). Furthermore, we and others have shown that in the mouse PCP model, total RNA samples from infected whole lungs as well as AMs derived directly from *P. jirovecii*-infected lungs express induced levels of Dectin-1 mRNA (16, 18). Taken together, the data support a critical role for Dectin-1 in innate immunity during PCP.

## Dectin-2

Dectin-2, another member of the CLR family, recognizes α-mannan/mannose structures on the cell wall of fungal organisms (39, 40). Dectin-2 has been shown to form heterodimers with the CLR Macrophage C-type lectin (MCL) leading to greater inflammatory responses then receptor alone when binding to α-mannans (41). We have shown that Dectin-2 can significantly bind live *Pneumocystis* organisms using a Dectin-2 Fc fusion protein and that this binding could be significantly reduced when the fungi were heat-treated (56^0^C for 1 hour) (12). These data suggest that Dectin-2 recognizes α-mannan/mannose structure(s) on the cell surface. Further experiments determined that Dectin-2 CLR may specifically bind a component(s) of native isolated Msg from *Pneumocystis*, as this *Pneumocystis* surface component significantly bound the Dectin-2 Fc fusion protein to a greater degree than the Fc control alone. Furthermore, RAW macrophages overexpressing full-length Dectin-2 demonstrated significantly greater binding of *Pneumocystis* organism compared to RAW cells treated with vector alone. Additionally, downstream spleen tyrosine kinase (Syk) activation following Dectin-2/Fcγ ligation was severely blunted in Dectin-2 deficient compared to wild type macrophages (22). Despite these data supporting strong interactions and activation of Dectin-2 signaling following interactions of this CLR with *Pneumocystis*, it was surprising when we observed that immunocompetent and immunosuppressed Dectin-2 deficient mice demonstrated statistically similar organism burdens and cytokine production compared to wild type mice (22). Therefore, taken together these data suggest a role for Dectin-2 in *Pneumocystis* host response events, but this CLR may play limited roles in controlling organism burden during PCP.

## Mincle/MCL

Macrophage inducible Ca^2+^-dependent lectin receptor (Mincle) was first described in fungal/host interactions with *Candida albicans* (42). This CLR is considered more promiscuous in its ligand binding, with known interactions with *Mycobacterium tuberculous* trehalose-6,6’-dimycolate (TDM) (43), human cholesterol crystals (44), and fungal α-mannose (45). Similar to Dectin-2, it has been shown that a Mincle Fc-fusion can significantly bind *Pneumocystis* Msg and that RAW macrophages overexpressing Mincle also bind more fungal organisms then the parent line alone. Furthermore, Mincle deficiency in macrophages also leads to decreased Syk phosphorylation, suggesting the importance of Mincle in the host downstream signaling proinflammatory response to *Pneumocystis* (16). However, unlike Dectin-2, in the PCP immunosuppressed model, Mincle knockout mice had considerably greater (~3X) *Pneumocystis* organism burdens then their wild type counterparts, suggesting the importance of this CLR in organism clearance during PCP (16).

Macrophage C-type lectin (MCL) is a CLR with considerably homology to Dectin-2 and Mincle. Like Mincle, it is has been shown to also bind α-mannose residues (46). Similar to what was reported for Mincle, MCL also binds *Pneumocystis* Msg in similar fashion when tested with the MCL Fc-fusion proteins as well as using whole organisms (12). One recent exciting finding was that silencing mRNA expression of both MCL and Mincle together in the RAW macrophage cell line prior to the overnight addition of *Pneumocystis* β-glucans and in the presence of Msg resulted in substantial reduction of TNFα secretion. Although individually silencing each CLR alone reduced TNFα considerably, their levels of inhibition did not achieve the synergistically reduced levels of the double silenced cell line (11). These data suggest the possible coregulation of the host immune response to *Pneumocystis* through Mincle-MCL CLR interdependent expression (47). Similar events have been described with these two receptors and the bacterial ligand mycobacterial cord factor (48). Future studies of the PCP mouse model in MCL/Mincle double deficient animals would be interesting to test the validity of this hypothesis.

## Mannose-Binding Lectin

Mannose-binding lectin (MBL) is a soluble CLR shown to be important in innate immunity against fungi through activation of complement and participating in phagocytosis. Ligands for MBL include N-acetylglucosamine linkages and mannose residues (49, 50). Studies to date report that MBL polymorphisms in HIV-patients confer more susceptibility to PJP (23). More recently it was determined that in another cohort of HIV-patients in Northern Thailand, MBL mutations resulting in low MBL expression had significantly higher incidence of PJP (24). Currently, potential *Pneumocystis* ligands for MBL are not yet known.

## Mannose Receptor

One of the first CLRs described to function as a myeloid receptor for *Pneumocystis* was the mannose receptor (MR) reported in the early 1990s. Ezekowitz and colleagues have shown that binding of *Pneumocystis* to AMs was competitively inhibited with mannose antagonists and that COS (CV-1 (simian) in Origin, and carrying the SV40 genetic material) (51) cells expressing human MR readily bound and engulfed *Pneumocystis* organisms, and that this phagocytosis required both transmembrane and cytoplasmic regions of the MR (29, 30). Later, O’Riordan et al. identified Msg as a ligand for MR, and utilizing a similar competitive assay with purified Msg as the soluble competitor, determined the importance of MR in mediating attachment of *Pneumocystis* to AMs (28). These studies led to a novel initial hypothesis that CLRs maybe used therapeutically in PJP (28). It was further shown that AMs from HIV positive individuals exhibited downregulated MR, potentially representing a contributing factor for impaired organism uptake by AMs that increases susceptibility to *Pneumocystis jirovecii* (27). From these observations, these researchers designed an MR-Fc fusion protein that, when incubated with *Pneumocystis*, increased phagocytic potential by greater than 8-fold, suggesting a potential clinical tool for treating HIV MR-downregulated AMs (26).

Further evidence beyond MR roles in phagocytic and endocytic functions was reported by Zhang et al. and linked NF-κB activation with MR. Sugar competition assays as well as targeted siRNA of MR, resulted in significant reductions in NF-κB nuclear translocation when challenged with *Pneumocystis*, linking the proinflammatory response and MR to the organism (25). Although these *in vitro* data suggest importance of MR in PCP, immunocompetent and immunosuppressed mouse models of PCP reported no significant differences comparing wild type and MR knockout animals. These authors therefore concluded that the absence of this receptor may be redundant and that other receptor(s) may compensate for the receptor absence (52).

## Other Non-Signaling Lectin Binding Proteins

### Surfactant Protein A

Surfactant protein A (SP-A), was the first pulmonary surfactant proteins associated with binding to *Pneumocystis*, with purified SP-A being shown to bind to the mannose rich Msg component of the organism (53). This lectin was found to be in significantly greater quantities in all AIDS-related pneumonias including PJP (33). Later it was determined that SP-A specifically enhanced the attachment of *Pneumocystis* organisms to rat AMs (32). Others have shown that in normal human AMs, the presence of SP-A on the surface of *Pneumocystis* correlates with decreased organism phagocytosis and maybe a contributor to the pathogenesis of PJP (31). Linke et al. was the first to show that immunosuppressed SP-A deficient mice exhibited higher organism burden and higher histological score (percentage of alveolar involvement). These data led these researchers to suggest that SP-A is indeed needed for organism burden control and modulated AM inflammatory responses to the organism (54).

### Surfactant Protein B

Studies regarding the role of another lung surfactant lectin, surfactant protein B (SP-B) are limited. Beers et al. showed that in the immunosuppressed mouse PCP model, SP-B is downregulated at both the mRNA and protein level. They concluded that this may be a pathogenic factor that the organism uses to prevent AMs from phagocytosing the organism (34).

### Surfactant Protein D

Our lab performed a number of studies in the early 1990s examining the role of surfactant protein D (SP-D) in *Pneumocystis* host response. These studies revealed a number of main findings. As with SP-A, SP-D also accumulates in the lung during PCP (55) and is important for AM binding (56). Furthermore, as with SP-A, SP-D was found to bind the Msg component of the *Pneumocystis* cell wall (57). SP-D can also undergo different states of polymerization, with an increase in these events leading to greater aggregation of SP-D (58). Analyzing the various states of this collectin, we found that higher dodecameric forms of the protein bound fungal organisms significantly greater than the trimeric configuration of the protein (57). Furthermore, it was demonstrated that SP-D accumulations are high in animal models of PCP as well as those individuals with PJP (59, 60). As with SP-A, SP-D was proposed as a means by which the organism avoids host killing (61). Interestingly however, in the CD4-depleted PCP mouse model, SP-D deficient animals surprisingly showed significantly higher organisms burdens along with higher lung inflammation scores, and lung weights. The authors suggests that the potential difference they noted compared to the previous studies where SP-D accumulates in the lung resulting in fungal aggregates and reduced organism clearance, might be due to due to various stages of the host response over the course of infections that differentially regulates both pro- and anti-inflammatory responses to the organism over time (62).

### Concluding Remarks

Innate immune receptors and lectins/collectins are an important part of the armature of the host defense against fungal pathogenic infections. Typically, they bind fungal mannoproteins or carbohydrates embedding and/or lining the fungal cell wall (63). The prototypic fungal cell wall is composed of chitin, α-glucans (both absent in *Pneumocystis*), β-1,3 and β-1,6 glucans, as well as a variety of low and high complex mannoproteins (64). CLRs and lectins bind their respective fungal ligands *via* their carbohydrate recognition domains (CRD) (65). Innate immune receptors bind their respective ligands resulting in downstream activation (*via* Syk phosphorylation, Protein kinase C alpha (PKCγ), Rat sarcoma virus (Ras)/Rapidly Accelerated Fibrosarcoma (Raf) for example), whereas collectins *via* binding the fungal ligand/organism help in macrophages phagocytosis and killing (66, 67). Currently, it is thought that there are more pattern recognition receptors (PRRs) (including CLRs and collectins) for fungi than any other organisms (68). This review is an attempt at highlighting the most current literature on carbohydrate recognition receptors involved in *Pneumocystis* organism/host cell interactions. Our understanding of the roles of specific lectins/receptors and the downstream inflammatory host response to *Pneumocystis* is still evolving. Host myeloid cells appear to have a high level of receptor/lectin redundancy in their identification and response to *Pneumocystis*, as individual absence of certain receptors is regarded as dispensable in regard to murine models of PCP (22, 52). In HIV/*Pneumocystis* co-infection, the role of these carbohydrate recognizing molecules is largely unknown because of lack of representative animal models. For example, it has been published that CLRs can promote protective anti-viral responses and aid in viral transmission (69). In this scenario of fungal/viral coinfection, CLR responses could be vastly different in the host immune signaling and/or organism uptake and killing then the single organism PCP CD4-depleted infection model. Future studies utilizing combination of *Pneumocystis* CLR/lectin ligands such as Msg/β-glucans and HIV envelope protein gp120 in *in vivo* assays with AMs and DCs might yield important early insights into the role this coinfection may play in the pathogenesis of HIV/PJP. In closing, this minireview summarizes our understanding of the current information on the CLRs/lectins linked with *Pneumocystis* and myeloid cell interactions.

## Author Contributions

This mini-review was written by TJK, EMC, and AHL. All authors contributed to the article and approved the submitted version.

## Funding

National Heart, Lung, and Blood Institute R01-HL62150-100% funding source.

## Conflict of Interest

The authors declare that the research was conducted in the absence of any commercial or financial relationships that could be construed as a potential conflict of interest.

## Publisher’s Note

All claims expressed in this article are solely those of the authors and do not necessarily represent those of their affiliated organizations, or those of the publisher, the editors and the reviewers. Any product that may be evaluated in this article, or claim that may be made by its manufacturer, is not guaranteed or endorsed by the publisher.
